# Anthropogenic nutrient loads and season variability drive high atmospheric N_2_O fluxes in a fragmented mangrove system

**DOI:** 10.1038/s41598-021-85847-6

**Published:** 2021-03-25

**Authors:** N. Regina Hershey, S. Bijoy Nandan, K. Neelima Vasu, Douglas R. Tait

**Affiliations:** 1 Department of Zoology, N.S.S. Hindu College, Changanassery, 686 102 India; 2 Department of Marine Biology, Microbiology and Biochemistry, School of Marine Sciences, Cochin University of Science and Technology, Cochin, 682 016 India; 3 Faculty of Environment and Science, Southern Cross University, Lismore, NSW 2480 Australia; 4 National Marine Science Centre, Southern Cross University, PO Box 4321, Coffs Harbour, NSW 2450 Australia

**Keywords:** Ecology, Biogeochemistry, Climate sciences, Ecology, Environmental sciences, Hydrology, Limnology

## Abstract

Fragmented mangroves are generally ignored in N_2_O flux studies. Here we report observations over the course of a year from the Mangalavanam coastal wetland in Southern India. The wetland is a fragmented mangrove stand close to a large urban centre with high anthropogenic nitrogen inputs. The study found the wetland was a net source of N_2_O to the atmosphere with fluxes ranging between 17.5 to 117.9 µmol m^−2^ day^−1^ which equated to high N_2_O saturations of between 697 and 1794%. The average dissolved inorganic nitrogen inputs (80.1 ± 18.1 µmol L^−1^) and N_2_O emissions (59.2 ± 30.0 µmol m^−2^ day^−1^) were highest during the monsoon season when the rainfall and associated river water inputs and terrestrial runoff were highest. The variation in N_2_O dynamics was shown to be driven by the changes in rainfall, water column depth, salinity, dissolved oxygen, carbon, and substrate nitrogen. The study suggests that fragmented/minor mangrove ecosystems subject to high human nutrient inputs may be a significant component of the global N_2_O budget.

## Introduction

Increasing anthropogenic terrestrial nitrogen (N) inputs and sewage pollution is regarded as one of the major cause for N enrichment in coastal wetlands. Coastal wetlands, estuaries and adjoining mangrove fringes which are in close proximity to human settlements can act as important sinks of terrestrial N where it can be stored and cycled. It is reported that 1% of reactive nitrogen entering estuaries are released as nitrous oxide (N_2_O) emissions^1^. N_2_O is a major contributor to stratospheric ozone depletion, has a lifetime of 118–131 years in the atmosphere^2^ and has about 300 times greater global warming potential than carbon dioxide (CO_2_)^3^. N_2_O is produced as an intermediate product during the denitrification process (heterotrophic NO_3_ reduction) and also as a by-product during the nitrification process (autotrophic oxidation of NH_4_ to NO_3_) and it is consumed during complete denitrification of N_2_O via dissimilatory reduction to N_2_^4^. Therefore, the ratio of production and consumption of N_2_O determines whether these coastal wetlands are sinks or sources of N_2_O. However, the production and consumption of N_2_O in a system is determined by several drivers such as temperature, pH, dissolved oxygen (DO), carbon (C) and hydrologic parameters in the environment^4^.

Mangroves increase the ecosystem value of coastal wetlands through their significant role in nutrient cycling, moderation of extreme weather events, supply of food and raw materials, and provision of breeding, spawning and nursery habitat for a variety of invertebrates and commercial fish species. However, the effectiveness of mangroves to meet these services is affected by the degree of anthropogenic disturbance. Recent studies have shown that tropical mangrove wetlands are under high risk from anthropogenic stresses particularly due to the extensive use of terrestrial N fertilizers causing dissolved inorganic nitrogen (DIN) enrichment^5,6^. Major mangrove ecosystems around the globe have been assessed for their contribution to atmospheric N_2_O budgets. However, despite our knowledge about the contribution of mangrove wetlands in the global N_2_O budget, uncertainties still exist due to lack of information about the impact of fragmented mangrove wetlands which are numerous in the tropical regions and particularly along the Indian coastline. As tropical areas receive disproportionately more rainfall than temperate areas, this likely leads to greater terrestrial N into coastal wetlands from nearby urban and agricultural areas. The high seasonal variability in rainfall distribution patterns in tropical areas may also drive large temporally variability in coastal N_2_O dynamics.

Along the Indian coast (7,516.6 km long^7^), mangrove cover an area of 497,500 ha^8^. The India state Kerala in southern India, was once rich in mangrove habitats, but the area of intact mangrove has drastically declined due to anthropogenic pressures such as urbanisation, burgeoning power projects and rapid industrialisation. It is estimated that the Ernakulum district in Kerala has 943 mangrove stands of which 880 (~ 93%) of them have an area less than 1 hectare. The contribution of these smaller mangrove systems is often ignored and their role in global nutrient cycling has yet to be adequately determined. As most of these mangrove stands are generally observed near the vicinity of human settlements, they can be critically influenced by the N loading there. Recent global studies suggest that aquatic environments influenced by intensive agricultural practices and industrial activities can act as source of N_2_O to the atmosphere^9–11^ and this predicted rise in terrestrial N concentrations can potentially triple the global N_2_O emissions from coastal wetlands^1^. However, Indian mangrove stands have been mostly ignored from the N input and output studies due to their relatively smaller area and footprint. The few studies that have taken place in Indian mangroves suggest that they are highly influenced by anthropogenic nutrient inputs and are significant sources of atmospheric N_2_O^12–14^. In light of the increasing anthropogenic N inputs, it is important to monitor and assess the potential of fragmented/minor mangrove stands for their contribution of N_2_O to the atmosphere.

In order to understand the contribution of fragmented/minor mangrove stands to atmospheric N_2_O emissions, this study aimed to assess the Mangalavanam Coastal Wetland (MCW) a fragmented minor mangrove stand adjacent to the city of Kochi, India over a one year period incorporating a range of distinct seasons. The N_2_O flux rates were calculated using three different k models^15–17^ to reduce the uncertainty in calculated N_2_O flux rates. The study also tested the relation between N substrates and N_2_O fluxes with temperature, pH, DO, and C loading of the wetland to determine the drivers of N_2_O dynamics.

### Study area

**Figure 1:**
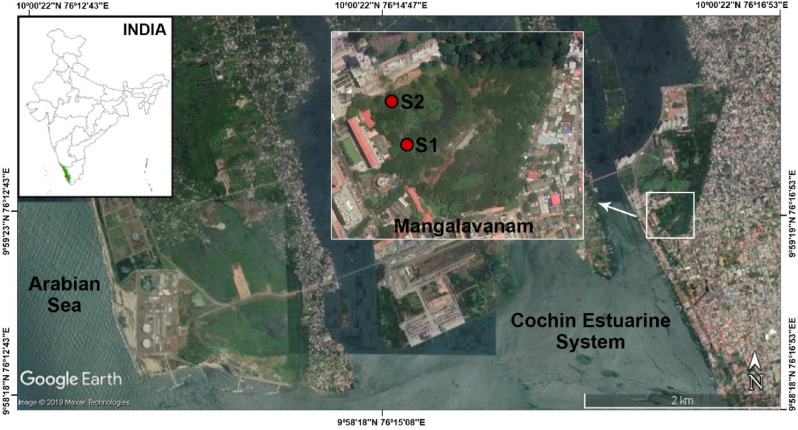
Map of Mangalavanam Coastal Wetland, Kerala, India depicting the study stations [source: Google earth pro V 7.3. (November 12, 2019). Kochi, Kerala. 9° 59′ 23.83″ N, 76° 16′ 26.74″ E, Eye alt 7.6 km. https://earth.google.com/web/search/Mangalavanam+Bird+Sanctuary,+High+Court+Road,+Ayyappankavu,+Kochi,+Kerala,+India/@9.9893564,76.2747626,4.86064735a,2322.96914379d,35y,240.09539944h,45t,0r/data=Cr0BGpIBEosBCiUweDNiMDgwZDU5Mjk0ZWQxZDU6MHhlMjcwYmIzMGIwZGY3OGYwGTUtDOyM-iNAIX5K37WVEVNAKlBNYW5nYWxhdmFuYW0gQmlyZCBTYW5jdHVhcnksIEhpZ2ggQ291cnQgUm9hZCwgQXl5YXBwYW5rYXZ1LCBLb2NoaSwgS2VyYWxhLCBJbmRpYRgCIAEiJgokCf0tW8UnikNAEZyxjdwYtjXAGXdPXswSvVxAISlSbeKAkktA].

The MCW is a semi-closed fragmented mangrove stand in the Ernakulam district along the south-west coast of India at 9° 59′ 23.83″ N and 76° 16′ 26.74″ E (Fig. 1). It receives tidal input through a narrow channel/feeder canal from the adjacent nutrient rich Cochin Estuarine System (CES), where eutrophication is of great concern^18^. This tidal wetland has a wet and maritime tropical climate with three distinct seasons: pre-monsoon (February to May), southwest monsoon (June to September, from here on called monsoon) and northeast monsoon (October to January) or post-monsoon. The wetland is relatively shallow (< 0.5 m) and is largely inundated during high tide flooding. The dominant mangrove species present in the MCW are *Rhizophora mucronata*, *Avicennia officinalis* and *Acanthus ilicifolius*. Mangrove associates such as *Acrostichum aureum, Derris trifoliate* and *Morinda citrifolia* are also common. Kochi is the most densely inhabited city in Kerala with an urban population of more than of 2.1 million in an area of 440 km^2^. The MCW receives nutrient input from the urban drainage systems of the adjacent human settlements. Due to government restrictions on sampling in the area, only two sites could be sampled, a site heavily influenced by upstream estuarine inputs (Site 1) and a relatively pristine site (Site 2).

## Results

### Physico-chemical attributes

**Figure 2:**
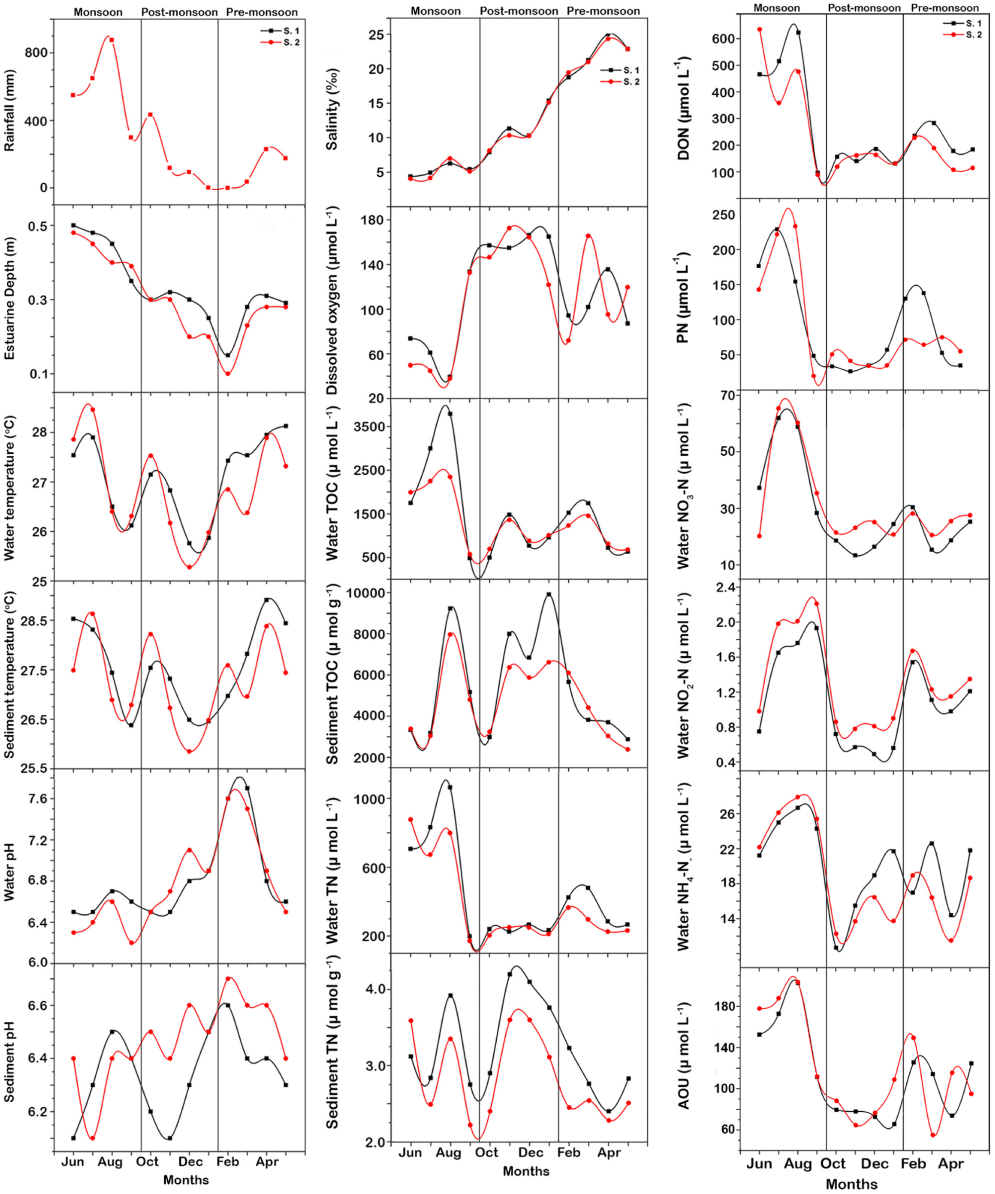
Variation in environmental parameters observed in the Mangalavanam Coastal Wetland, India during the study.

The area received 3469 mm of rainfall over the study period, with 68.5% falling during the monsoon season (2376 mm) (Fig. 2, Table 1). The mean wind speed of the study area was 2.4 ± 0.4 m s^−1^, with monthly averages ranging from 2.0 m s^−1^ in April to 3.2 m s^−1^ in June. The wetland depth was generally shallow, with a mean depth of 0.3 ± 0.8 m and ranged from 0.1 m in February to 0.5 m in June. The mean annual water and sediment temperature was 27.0 ± 0.9 °C and 27.4 ± 0.8 °C, respectively. The mean mixo-mesohaline to mixo-polyhaline salinity was 12.3 ± 7.2 ‰, with significant differences between seasons (*f* = 107.20, *ρ* ˂ 0.001). The mean DO concentrations in the water column was 112.2 ± 45.6 µmol L^−1^ and decreased from the post-monsoon season (156.1 ± 16.0 µmol L^−1^) to the pre-monsoon season (109.0 ± 30.1 µmol L^−1^), and to the monsoon season (71.6 ± 39.8 µmol L^−1^). The annual mean DO saturation was 229.2 ± 11.2% and AOU was 117.0 ± 45.7 µmol L^−1^ with both varying significantly between seasons (*f* = 32.00, *ρ* ˂ 0.001 and *f* = 16.36, *ρ* ˂ 0.001, respectively). The pH of the water column over the year ranged from 6.5 to 7.6 with a mean of 6.8 ± 0.3, while in the sediments it ranged from 6.1 to 6.7. There was a significant difference in pH in the water column between seasons (*f* = 9.07, *ρ* = 0.002) and in sediment pH between stations (*f* = 4.72, *ρ* = 0.043).

**Table 1:** Variation in selected environmental parameters observed in the Mangalavanam Coastal Wetland, India during the study.

Parameters	Monsoon	Post-monsoon	Pre-monsoon
Rainfall (mm)	594.0 ± 239.7	162.4 ± 188.3	110.8 ± 109.4
Wind speed (m s^−1^)	2.9 ± 0.3	2.2 ± 0.1	2.2 ± 0.3
Estuarine depth (m)	0.4 ± 0.1	0.3 ± 0.1	0.2 ± 0.1
Water temperature (°C)	28.2 ± 0.5	26.6 ± 0.6	28.8 ± 0.8
Sediment temperature (°C)	29.6 ± 0.9	26.9 ± 0.8	27.8 ± 0.7
Salinity (‰)	5.2 ± 1.0	11.1 ± 2.8	21.9 ± 2.2
DO (µmol L^−1^)	71.6 ± 39.8	156.1 ± 16.0	109.0 ± 30.1
DO saturation (%)	236.6 ± 7.5	235.4 ± 3.7	215.6 ± 4.7
AOU (µmol L^−1^)	165.1 ± 36.9	79.3 ± 14.2	106.7 ± 30.5
Water pH	6.6 ± 0.1	6.7 ± 0.2	7.0 ± 0.4
Sediment pH	6.3 ± 0.2	6.4 ± 0.2	6.5 ± 0.1
Water TN (µmol L^−1^)	640.5 ± 289.7	235.5 ± 20.8	322.1 ± 92.3
Water DN (µmol L^−1^)	487.4 ± 226.5	196.3 ± 25.2	244.4 ± 63.6
Water PN (µmol L^−1^)	153.1 ± 81.1	39.2 ± 10.1	77.7 ± 36.9
Water DON (µmol L^−1^)	407.3 ± 213.3	148.5 ± 22.0	190.0 ± 59.5
Water DIN (µmol L^−1^)	80.1 ± 18.1	47.8 ± 10.4	54.4 ± 12.7
Water NH_4_ (µmol L^−1^)	25.7 ± 2.4	19.1 ± 4.7	18.9 ± 4.9
Water NO_2_ (µmol L^−1^)	1.7 ± 0.5	0.7 ± 0.2	1.3 ± 0.2
NO_3_ (µmol L^−1^)	52.7 ± 15.2	27.9 ± 5.6	34.2 ± 7.5
Sediment TN (µmol g^−1^)	3.0 ± 0.6	3.5 ± 0.6	2.6 ± 0.3
Water TC (µmol L^−1^)	5255.6 ± 1995.5	3048.3 ± 704.3	3444.8 ± 921.2
Water TOC (µmol L^−1^)	2023.5 ± 1120.7	956.3 ± 329.7	1099.9 ± 440.3
Water TIC (µmol L^−1^)	3232.1 ± 874.9	2092.1 ± 374.63	2344.9 ± 480.9
Sediment TOC (µmol g^−1^)	5013.1 ± 2368.9	6230.3 ± 2292.4	4000.8 ± 1323.8
Dissolved N_2_O (nM)	80.2 ± 26.6	74.6 ± 14.0	73.2 ± 11.7
N_2_O saturation (%)	1103.0 ± 356.6	1022.6 ± 190.8	1137.9 ± 194.7
N_2_O flux, Wanninkhof, 1992 (μmol m^−2^ day^−1^)	59.2 ± 30.0	28.0 ± 5.9	30.2 ± 6.9
N_2_O flux, Raymond and Cole, 2001 (μmol m^−2^ day^−1^)	112.6 ± 48.4	74.7 ± 14.9	78.5 ± 12.1
N_2_O flux, Wanninkhof and McGillis, 1999 (μmol m^−2^ day^−1^)	16.0 ± 9.4	5.5 ± 1.3	6.2 ± 2.2

### Carbon and nitrogen measurements

The total nitrogen (TN) concentration in the water column ranged from 172.1 to 877.1 µmol L^−1^, with an annual mean of 399.4 ± 244.8 µmol L^−1^ and in the sediments it ranged from 158.6 to 301.4 µmol g^−1^ (Table 1). The mean TN in the water column was highest during the monsoon season and the lowest during post-monsoon season, while in sediments the maximum concentration was observed during the post-monsoon season and the minimum during the pre-monsoon season. Dissolved nitrogen (DN) contributed 66 to 88% of the observed TN with an annual mean of 322.1 ± 182.0 µmol L^−1^ and varied significantly on temporal basis (*f* = 9.16, *ρ* = 0.002). Dissolved inorganic nitrogen constituted 12–41% of the DN concentrations with an annual mean of 60.7 ± 18.1 µmol L^−1^, while DON was 58–88% of the DN concentration with an annual mean of 248.6 ± 168.9 µmol L^−1^. The DIN and DON concentrations in the water column varied significantly on seasonal basis (*f* = 17.15, *ρ* ˂ 0.001 and *f* = 8.26, *ρ* = 0.003, respectively) with higher DIN and DON in the monsoon season. NO_3_ was the major constituent of DIN, with a mean of 62 ± 8% of DIN. NO_3_ was followed by NH_4_ and NO_2_, constituting about 36 ± 8% and 2 ± 1%, of the DIN respectively. There was a significant difference in water column NH_4_ (*f* = 8.89, *ρ* = 0.002), NO_2_ (*f* = 15.47, *ρ* < 0.001) and NO_3_ (*f* = 11.89, *ρ* = 0.001) seasonally. Particulate nitrogen (PN) in the water column ranged from 20.1 µmol L^−1^ to 232.9 µmol L^−1^ during the study period and varied significantly between seasons (*f* = 8.76, *ρ* = 0.002). The total carbon (TC) concentration in the water column ranged from 2473.3 to 7391.7 µmol L^−1^ (average 3916.3 ± 1453.0 µmol L^−1^) with total organic carbon (TOC) contributing 33 ± 10% of the observed TC concentrations (range from 484.2 to 3792.5 µmol L^−1^) which varied significantly on seasonal basis (*f* = 4.63, *ρ* = 0.02). In the sediment, TOC concentrations ranged between 2877.5 µmol g^−1^ to 9911.7 µmol g^−1^ over the study period. In the water column, the mean TOC concentration was highest during the monsoon season and lowest during the post-monsoon season, while in sediments, the maximum concentration was observed during the post-monsoon season (6230.3 ± 2292.4 µmol g^−1^) and the minimum concentration was observed during the pre-monsoon season (4000.8 ± 1323.8 µmol g^−1^). The total inorganic carbon was the major constituent of TC in the water column, with an annual mean of 2556.4 ± 771.6 µmol L^−1^, ranged from 1578.3 µmol L^−1^ in July to 4605.0 µmol L^−1^ in November and varied significantly between seasons (*f* = 7.57, *ρ* = 0.003).

### Nitrous oxide dynamics

**Figure 3:**
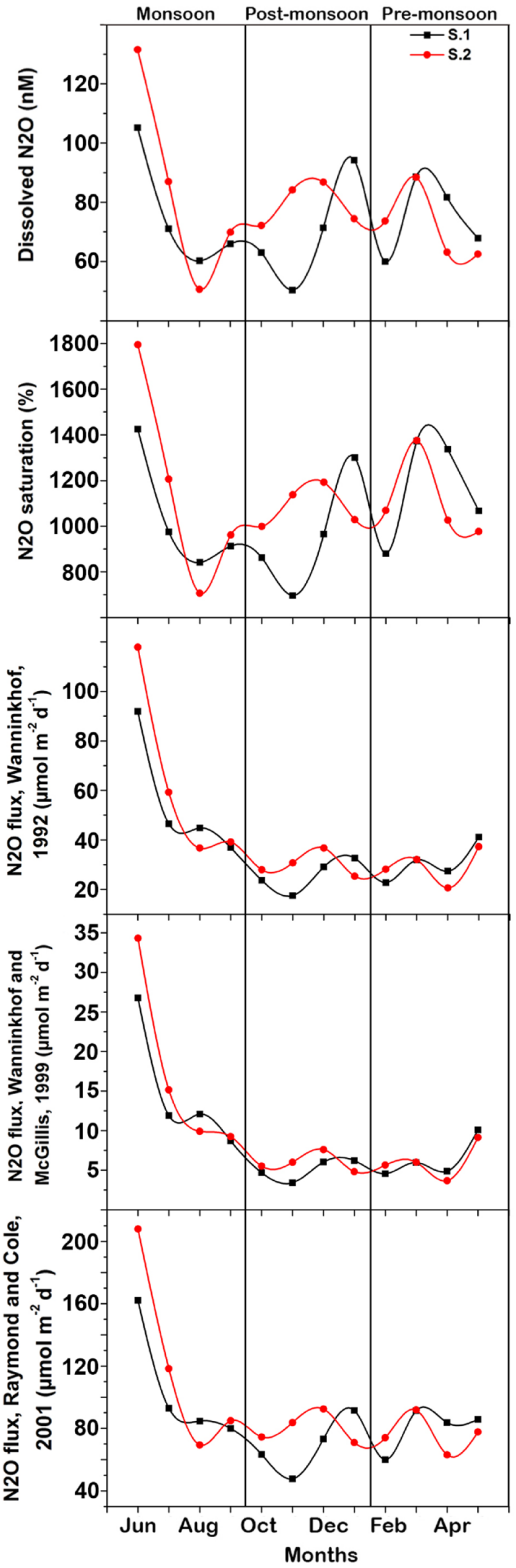
Variation in N_2_O dynamics observed in the Mangalavanam Coastal Wetland, India during the study.

The dissolved N_2_O concentration in the water column of the MCW ranged from 50.3 nM in November to 131.5 nM in June (Fig. 3, Table 1). The mean percentage saturation of N_2_O was 1088 ± 252% and ranged from 696 to 1794%, with relatively little seasonal variations. Using Wanninkhof^15^ k values, N_2_O fluxes ranged from 17.5 to 117.9 μmol m^−2^ day^−1^, with an annual mean of 39.1 ± 22.6 μmol m^−2^ day^−1^. For comparisons sake, the flux calculations of Raymond and Cole^17^, N_2_O fluxes ranged from 47.7 to 207.9 μmol m^−2^ day^−1^, with an annual mean of 88.6 ± 33.6 μmol m^−2^ day^−1^ and Wanninkhof and McGillis^16^ N_2_O fluxes ranged from 3.4 to 34.3 μmol m^−2^ day^−1^, with an annual mean of 9.26 ± 7.27 μmol m^−2^ day^−1^. Using Wanninkhof^15^ and Wanninkhof and McGillis^16^, the N_2_O flux varied significantly on seasonal basis (*f* = 6.53, *ρ* = 0.007 and* f* = 7.55, *ρ* = 0.004, respectively), while no significant variation was observed with the N_2_O flux that was calculated using Raymond and Cole^17^ calculated fluxes (*f* = 3.43, *ρ* = 0.055). The N_2_O flux calculated using the three models was highest during the monsoon season and lowest during the post-monsoon season.

## Discussion

This study found that the MCW fragmented mangrove stand is a C and N rich environment which favours substantial N_2_O production and its release into the atmosphere. The N pool and the atmospheric flux of N_2_O in the MCW (39.1 ± 22.6 μmol m^−2^ day^−1^) was found to be higher than that reported in the adjacent CES (11.4 ± 6.9 μmol m^−2^ day^−1^) where there are significant anthropogenic N inputs^19^. In the Bhitarkanika mangrove on the east coast of India^12^, high N_2_O emissions were observed (5–113.38 μmol m^−2^ day^−1^) driven by high anthropogenic nutrient loading. In other Indian mangroves–Muthupet mangrove in southern India, comparatively low N_2_O emissions (9.3–18.18 μmol m^−2^ day^−1^) were reported even though the mangrove is subjected to a range of anthropogenic inputs including aquaculture (shrimp-farming effluent) and seasonal agricultural run-off^13^. Our observations were also in the range of previous studies from the large eutrophic Pearl River Estuary in China (37 ± 15 μmol m^−2^ day^−1^) which receives extensive urban, industrial and agricultural run-off^10^.

In a range of pristine mangroves in Australia^20^, the uptake of N_2_O has been reported with an uptake rate of 1.52 ± 0.17 μmol m^−2^ day^−1^. However, the relative pristine mangrove site selected in our study (Site 2) was supersaturated with dissolved N_2_O (1122.8 ± 266.9%) and acted as atmospheric source of N_2_O (41.0 ± 26.1 μmol m^−2^ day^−1^). A recent study on N_2_O dynamics from four southern hemisphere subtropical estuaries^11^ (an urbanised estuary, mixed, urban and agricultural estuary and pristine estuary) reported that the N_2_O saturation ranged from 77.7 to 381.5% with highest saturations in the pristine estuary and lowest in the agricultural estuary, which was much lower than the range seen in this study (696.7–1794.3%). The study also reported that estuaries in the two urbanised catchments had the highest mean N_2_O flux rates during summer (18.8 ± 11.6 μmol m^−2^ day^−1^ and 18.7 ± 12.8 μmol m^−2^ day^−1^, respectively) with the pristine estuary having a summer flux rate of 5.1 ± 6.9 μmol m^−2^ day^−1^^11^. Other studies from estuaries in urbanised catchments have reported low mean N_2_O flux rates, with 4.0 μmol m^−2^ day^−1^ reported in the Yarra River in Melbourne^21^, and 7.3 μmol m^−2^ day^−1^ in the Brisbane River^22^. The high flux rates observed here are likely a result of high rainfall in the tropics driving higher freshwater inputs, terrestrial runoff and the enhanced anthropogenic N enrichment compared to other estuaries cited above. The pristine site (Site 2) also received tidal inputs from the nutrient enriched CES which would lead to higher N_2_O concentrations in the water column compared to Site 1 that was heavily influenced by upstream estuarine inputs. Also, the water column of Site 1 drains out completely into the CES during low tides; however the water column depth of Site 2 was less affected during low tides which likely reduced the export of N to adjacent waters.

Significant uncertainties exist in global N_2_O budget due in part to the differences in methods used in calculating flux rates. Two meta‐analyses published in the past 20 years highlight that the lack of direct measurements of k significantly hampers the ability to parameterize air‐water gas exchange in estuaries and that care should be given when choosing values of k, with respect to location‐dependent controls on gas exchange^17,23^. Here we offer a comparison of the different flux rates calculated using three different and commonly used k value models (Table 1) and the relationship of various measured parameters to N_2_O flux rates calculated using three models (Table 2). The parameterization of Raymond and Cole^17^ is widely used for rivers and streams, where the turbulence reaching the air‐water interface is chiefly generated by friction with the bottom. The parameterization of Wanninkhof and McGillis^16^ is best used for open water bodies but can underestimate N_2_O flux rates where wind speeds are low such as sheltered mangrove systems. The k value model of Wanninkhof^15^ is also commonly used and uses the Schmidt number of the water related to viscosity and with its exponent reflecting the surface layer’s rate of turbulent renewal. As water column depth, wind speed, and current velocity act as main drivers of these k models and influences turbulence at the air–water interface, in shallow flowing ecosystems^24,25^, such as in mangroves, we felt the k value model of Wanninkhof^15^ best fits for the study.

**Table 2:** Relationship of N_2_O flux calculated using different models to other environmental parameters observed in the Mangalavanam Coastal Wetland, India during the study.

Pearson correlation			
Parameters	N_2_O flux Wanninkhof (1992)	N_2_O flux Raymond and Cole (2001)	N_2_O flux Wanninkhof and McGillis (1999)
Rainfall	r = 0.463, ρ = 0.023	Not significant (N.S.)	r = 0.501, ρ = 0.013
Wind speed	r = 0.827, ρ ˂ 0.001	r = 0.634, ρ = 0.001	r = 0.790, ρ ˂ 0.001
Estuarine depth	r = 0.670, ρ ˂ 0.001	r = 0.567, ρ = 0.004	r = 0.696, ρ ˂ 0.001
Salinity	r = − 0.484, ρ = 0.017	N.S	r = − 0.505, ρ = 0.012
DO	r = − 0.516, ρ = 0.010	N.S	r = − 0.554, ρ = 0.005
AOU	r = 0.528, ρ = 0.009	N.S	r = 0.570, ρ = 0.004
TC (water)	r = 0.467, ρ = 0.022	N.S	r = 0.494, ρ = 0.014
TIC (water)	r = 0.481, ρ = 0.017	N.S	r = 0.504, ρ = 0.012
TN (water)	r = 0.664, ρ ˂ 0.001	r = 0.573, ρ = 0.003	r = 0.690, ρ ˂ 0.001
DN (water)	r = 0.701, ρ ˂ 0.001	r = 0.611, ρ = 0.002	r = 0.726, ρ ˂ 0.001
NH_4_ (water)	N.S	N.S	r = 0.434, ρ = 0.034
NO_3_ (water)	r = 0.617, ρ = 0.001	r = 0.523, ρ = 0.009	r = 0.638, ρ = 0.001
DIN (water)	r = 0.612, ρ = 0.001	r = 0.496, ρ = 0.014	r = 0.640, ρ = 0.001
PN (water)	r = 0.482, ρ = 0.017	r = 0.400, ρ = 0.053	r = 0.505, ρ = 0.012
DON (water)	r = 0.700, ρ ˂ 0.001	r = 0.614, ρ = 0.001	r = 0.725, ρ ˂ 0.001
Dissolved N_2_O	r = 0.654, ρ ˂ 0.001	r = 0.813, ρ ˂ 0.001	r = 0.590, ρ = 0.002
N_2_O saturation	r = 0.654, ρ = 0.001	r = 0.813, ρ ˂ 0.001	r = 0.590, ρ = 0.003

The study observed a relationship between N_2_O dynamics and the availability of nitrogen species (Table 2). Concentrations of TN, DN, DON, NO_3_, NH_4_ and NO_2_ in the study area were high and at the high end of the range of concentrations seen in other highly polluted estuaries^26^. MCW is located in the heart of the densely populated Kochi city and has several sewage inlets from surrounding human settlements. This significantly increases the availability of N in both sediments and the water column leading to higher N_2_O production in the study area. Recent studies have shown that sewage influxes increase N_2_O production^19,22^. The release of nitrogenous compounds through the excreta of water-birds can also cause N enrichment in mangroves^26^ and the abundant water bird population of MCW would also lead to higher nitrogen loads in the study sites.

The MCW also receives significant N inputs through the narrow channel from CES that receives substantial fresh water discharge (approx. 1.2 × 10^10^ m^3^ year^−1^) from the six major rivers in the area (Pamba, Manimala, Achancoil, Meenchil, Periyar and Muvattupuzha Rivers) during the monsoon season^27,28^ and also receives inputs of approximately 260 m^3^ of domestic and 104 × 10^3^ m^3^ of industrial waste discharges daily, most of which are unprocessed^29,30^. The CES (and subsequently the MCW) receives high inputs of N from the surrounding agricultural areas which are flushed into receiving waters (both from surface runoff and groundwater inputs). There were significant relationships between rainfall distribution pattern and NH_4_ (r = 0.415, ρ = 0.044), NO_2_ (r = 0.489, ρ = 0.015), NO_3_ (r = 0.755, ρ = 0.000) concentrations and N_2_O flux calculated using Wanninkhof^15^ (Table 2). Similar observations of increased N loads and associated N_2_O fluxes due to rainfall have been reported in the eutrophic Taihu Lake in China^31^. The high resident time of the water column in the study lead to greater accumulation of N in the water column and longer processing times of N before being exported which likely influence N_2_O production and flux rates in the study area.

High N and organic carbon concentrations have been shown to accelerate the nitrification–denitrification processes^31–33^ and lead to higher N_2_O production although C and N concentrations did not show a clear relationship to dissolved N_2_O concentration in the MCW. Non-significant relationships between dissolved N_2_O concentration and DIN have been observed in other recent studies^34–36^. However, during this study significant relationships were observed between TC, TIC, TN, DN, DIN, NO_3_, NH_4_, DON, PN and N_2_O flux calculated using Wanninkhof^15^ (Table 2). This indicates a consistent relationship existed between N, C inputs and N_2_O outputs. Significant relationships between DIN inputs and N_2_O fluxes have also been shown in the nearby CES in India^19^, rivers^37^ and estuaries globally^38^. Positive relationships were also observed between N_2_O and NH_4_ and NO_3_ + NO_2_ during the summer dry season, while during winter, N_2_O saturation was strongly correlated to NO_3_ + NO_2_ but not with NH_4_^11^.

The significant positive correlation between NO_3_ and NH_4_ concentrations to N_2_O flux calculated using Wanninkhof^15^ and strong negative correlation between DO and NH_4_ (r = − 0.558, ρ = 0.005), NO_2_ (r = − 0.586, ρ = 0.003) and NO_3_ (r = − 0.788, ρ ˂ 0.001) suggest high N_2_O flux rates produced through nitrification. However, lower DO during the monsoon months along with higher availability of NO_3_ in the presence of high organic carbon loading likely promotes denitrification increasing N_2_O production and its fluxes. Several studies suggest that estuaries receiving higher N inputs generally show oxygen depletion, which in turn triggers denitrification^34,39,40^. Increasing anthropogenic nitrogen inputs to the mangrove sediments along with periodic tidal flooding can generate anoxic conditions in the sediment; thereby further enhancing denitrification^41^. This process along with nitrification could significantly contribute to nutrient turnover and N_2_O production in mangroves. However, the magnitude at which a wetland can act as source of N_2_O cannot be explained solely based on N loads as the magnitude of N_2_O flux depends on both denitrification-nitrification rates (how efficiently N is cycled) and N_2_O reduction rates (how efficiently N_2_O is reduced to N_2_)^31^.

The highest dissolved N_2_O concentrations and saturations were observed during the pre-monsoon season when temperatures were highest, while the N_2_O flux rates were highest during monsoon season. However, there was no significant relationship between water column and sediment temperatures and N_2_O concentrations, saturations and flux rates. Recent studies suggest that higher temperatures during summer seasons (pre-monsoon) can enhance microbial activity favouring N_2_O production^9^, while the reduced freshwater discharge during summers can increase the resident time of the water column, leading to greater accumulation of N_2_O in water and reducing the N_2_O loss to the atmosphere due to the decrease in gas transfer velocity^42^.

The shallow depth profile of the MCW likely promoted easier diffusion of oxygen molecules into the water column and to the sediments, favouring nitrification. This can drive higher dissolved N_2_O concentrations and its saturation during the pre-monsoon than the monsoon and post-monsoon seasons. A recent report suggests that the shallow bathymetry of the CES favoured easier exchange of N_2_O that was produced in the estuarine sediment and water column to the atmosphere^19^. A positive correlation between water column depth and N_2_O flux was observed using all models (Table 2). The deepest water column depth was mainly observed during monsoon season when the C and N loads were highest; there was low DO saturation and wind speeds were highest. However the mean water column depth during the monsoon season remained shallow (less than 0.5 m) even though it had the highest range in depths (0.3–0.5 m). Combined with the higher wind speeds during the monsoon season, this resulted in higher N_2_O fluxes rates. The significant negative correlation between salinity and N_2_O flux calculated using Wanninkhof^15^ was likely driven by either nutrient rich seawater returned from the CES or the reduced solubility of gases in saline conditions.

During the study, DON was the major constituent of the N and signified that the N enrichment in the study area was mainly through organic matter inputs or oceanic inputs where DON is the most prevalent form of N. As the major source of organic carbon in the study area was likely from mangrove litter which is generally nitrogen deficient^43,44^, the positive correlation of TC to DON in the water column (r = 0.870, ρ < 0.001) suggests that the study area receives significant amount of organic inputs from allochthonous sources, particularly with high N concentrations. The significant positive correlation between TC and TIC in the water column and N_2_O flux indicates that the N_2_O production and higher fluxes occurs in N rich environments with regard to C availability. However, the study failed to explain the relationship of TOC to N_2_O production and fluxes. A recent study in the tropical estuaries of north-western Borneo^45^ also failed to explain the significant correlation between DOC and N_2_O concentrations. Although temperature and pH are important factors affecting N_2_O production, no clear relationship was found between these variables and dissolved N_2_O concentrations and fluxes. Several studies also suggest that the influence of water temperature on the denitrification rate may vary between regions and seasons^31,46,47^.

As the MCW is a fragmented mangrove, it’s atmospheric contribution of N_2_O calculated using Wanninkhof^15^ is quite small (ranging from 1.1 ± 0.2 × 10^6^ μmol day^−1^ during post-monsoon season to 2.4 ± 1.2 × 10^6^ μmol day^−1^ during monsoon season) and on its own may not be significant. However considering the numerous smaller mangrove stands that exist all over the tropics, fragmented mangroves may represent a significant source of N_2_O to the atmosphere. For example, the Ernakulam district in the state Kerala alone has about 933 minor mangrove stands, covering a total area of 206 hectares. This suggests that fragmented/minor mangrove stands may have an as yet unquantified but significant influence on atmospheric N_2_O budgets and atmospheric warming into the future.

## Conclusion

The net N_2_O flux from MCW suggest that it is a source of atmospheric N_2_O, however due to its small area of coverage its contribution to atmospheric N_2_O is minor. The high precipitation rates in the study, through terrestrial runoff and river water discharge and high N inputs, influenced N_2_O flux rates particularly during monsoon season. The study suggests that when taken as whole, fragmented/minor mangroves which are abundant in tropical regions, need to be critically assessed and protected from further anthropogenic loading. Further studies in a range of different geologic and hydrologic conditions will help to include this potentially significant ecosystem type in global N_2_O budgets. The study highlights that N_2_O flux rates were dependant on the availability of DIN as well as salinity, DO, estuarine depth, rainfall, wind speed and availability of carbon.

## Materials and methods

Field work was carried out over a period of 12 months from June 2014 to May 2015 with observations made during the morning hours on a monthly basis. Rainfall data was obtained from the Indian Meteorological Department. The depth of the wetland was measured by lowering a graduated weighted rope until it touched the top of the sediments. Transparency was measured using a 20 cm diameter Secchi disc^48^. Triplicates of nutrient samples were collected from surface waters at each site. Temperature, salinity and pH were measured using an Eutech water quality analyser (CyberScan Series SCD 650). Water samples for DO were taken in 60 ml glass bottles fixed with 0.5 ml each of Winkler reagents and titrated against sodium thiosulphate using visual endpoint detection^49^. Apparent oxygen utilisation (AOU) was calculated as outlined by Garcia and Gordon^50^, using the measured DO concentration. Dissolved inorganic nitrogen (DIN, which includes NH_4_, NO_2_, and NO_3_) were analysed spectrophotometrically following standard procedures^51^.

Water samples for total organic carbon (TOC) and total inorganic carbon (TIC), total nitrogen (TN), dissolved nitrogen (DN) and dissolved N_2_O were collected in 120 ml glass bottles. The samples were fixed using saturated mercuric chloride (0.6 ml/120 ml) to arrest microbial activity^52^. An aliquot of each sample was filtered through a 25 mm GF/F filter and the filtrate was collected for the measurement of DN, while the remainder of the sample was analysed for TOC, TIC and TN by wet combustion method using a TOC elemental analyser (Multi N/C 2100 S Analytik jena). Particulate nitrogen (PN) and dissolved organic nitrogen (DON) concentrations in the water column were calculated by subtracting DN from TN and DIN from DN, respectively.

Sediment samples were obtained using a van-Veen grab sampler (area 0.04 m^2^) with a glass corer (3 cm diameter) inserted into each grab sample. As the surface sediment contained mangrove litter, samples were collected at a depth of 2 cm from the surface of the sediment and sieved (usually < 2 mm), dried at 60 °C for 24–72 h and ground to a fine powder. An aliquot of the dried sediment sample was acidified using 1 M hydrochloric acid to remove the inorganic carbon present in the sample^53^. The acidified samples were then washed with distilled water, dried and ground to powder. The samples were then analysed for TOC using dry combustion method (TOC elemental analyser Multi N/C 2100 S, Analytik jena). The other part of the sample that was not treated with acid was used to measure TN in the sediment using Pyro-cube IRMS.

Dissolved N_2_O was determined by the multiple phase equilibration technique^54^.

The N_2_O water to air exchange fluxes (*f*) were computed using:

1 $$f = {\text{ k}{\alpha}}_{{}} \left( {{\text{C}}_{{\text{w}}} - {\text{ C}}_{{\text{a}}} } \right),$$

where k is the gas transfer coefficient of N_2_O^23,55^, α is the solubility coefficient of N_2_O calculated using temperature and salinity^56^, Cw is the water column N_2_O partial pressure and Ca is the local measured atmospheric value for N_2_O (323 ppb as recently reported)^57^. To provide a comparison of different k values, the wind parameterization k models of Wanninkhof^15^, Wanninkhof and McGillis^16^ and Raymond and Cole^17^ were calculated. Wind speed data (10 m height) were obtained from ERA interim, European Centre for Medium Range Weather Forecast (ECMWF) with a data resolution of ~ 80 km from the study site.

SPSS *v*16 (Statistical Programme for Social Sciences *v*16) was used for Pearson’s correlation analyses and the two-way analysis of variance (ANOVA) and Origin Pro 8.5 used to plot the graphs.

## Data Availability

Most of the data generated during the current study is graphically represented in the manuscript. The datasets generated during and/or analysed during the current study are also available from the corresponding author on reasonable request.

## References

[1] Kroeze C, Dumont E, Seitzinger SP (2005). New estimates of global emissions of N_2_O from rivers and estuaries. Environ. Sci..

[2] Ciais, P. *et al.* Carbon and other biogeochemical cycles. In *Climate Change 2013: The Physical Science Basis. Contribution of Working Group I to the Fifth Assessment Report of the Intergovernmental Panel on Climate Change* 465–570 (Cambridge University Press, 2014).

[3] Forster, P. *et al.* Changes in atmospheric constituents and in radiative forcing. Chapter 2. In *Climate Change 2007. The Physical Science Basis* (2007).

[4] Butterbach-Bahl K, Baggs EM, Dannenmann M, Kiese R, Zechmeister-Boltenstern S (2013). Nitrous oxide emissions from soils: How well do we understand the processes and their controls?. Philos. Trans. R. Soc. B..

[5] Reis CRG, Nardoto GB, Oliveira RS (2017). Global overview on nitrogen dynamics in mangroves and consequences of increasing nitrogen availability for these systems. Plant Soil..

[6] Rao K, Priya N, Ramanathan AL (2019). Impacts of anthropogenic perturbations on reactive nitrogen dynamics in mangrove ecosystem: Climate change perspective. J. Clim. Change.

[7] Centre for Coastal Zone Management and Coastal Shelter Belt, Ministry of Environment, Forests and Climate change, Govt. of India http://iomenvis.nic.in/index2.aspx?slid=758&sublinkid=119&langid=1&mid=1 (2017).

[8] FSI. India State of Forest Report. 2019. Forest Survey of India, Ministry of Environment and Forests, Dehradun (2019).

[9] Borges AV (2018). Effects of agricultural land use on fluvial carbon dioxide, methane and nitrous oxide concentrations in a large European river, the Meuse (Belgium). Sci. Total Environ..

[10] Lin H (2016). Spatiotemporal variability of nitrous oxide in a large eutrophic estuarine system: The Pearl River Estuary, China. Mar. Chem..

[11] Reading MJ (2020). Land use drives nitrous oxide dynamics in estuaries on regional and global scales. Limnol..

[12] Chauhan R, Ramanathan AL, Adhya TK (2008). Assessment of methane and nitrous oxide flux from mangrove along Eastern coast of India. Geofluids.

[13] Krithika, K., Purvaja, R. & Ramesh, R. Fluxes of methane and nitrous oxide from an Indian mangrove. *Curr. Sci.* **94**, 218224, https://www.jstor.org/stable/24101861 (2008).

[14] Fernandes SO, LokaBharathi PA, Bonin PC, Michotey VD (2010). Denitrification: An important pathway for nitrous oxide production in tropical mangrove sediments (Goa, India). J. Environ. Qual..

[15] Wanninkhof R (1992). Relationship between wind speed and gas exchange over the ocean. J. Geophys Res..

[16] Wanninkhof R, McGillis WM (1999). A cubic relationship between gas transfer and wind speed. Geophys. Res. Lett..

[17] Raymond PA, Cole JJ (2001). Gas exchange in rivers and estuaries: Choosing a gas transfer velocity. Estuaries.

[18] Hershey, R. N., Nandan, S. B. & Vasu, N. K. Trophic status and nutrient regime of Cochin estuarine system, India*. Indian J. Mar. Sci.* **49**(08), 2582–6727 http://nopr.niscair.res.in/handle/123456789/55309 (2020).

[19] Hershey RN (2019). Nitrous oxide flux from a Tropical estuarine system (Cochin estuary, India). Reg. Stud. Mar. Sci..

[20] Maher DT, Sippo JZ, Tait DR, Holloway C, Santos IR (2016). Pristine mangrove creek waters are a sink of nitrous oxide. Sci. Rep..

[21] Tait DR (2017). Greenhouse gas dynamics in a salt-wedge estuary revealed by high resolution cavity ring-down spectroscopy observations. Environ. Sci. Technol..

[22] Wells NS (2018). Estuaries as sources and sinks of N_2_O across a land use gradient in subtropical Australia. Glob. Biogeochem. Cycles..

[23] Upstill-Goddard RC (2006). Air–sea gas exchange in the coastal zone. Estuar Coast Shelf Sci..

[24] Zappa CJ, Raymond PA, Terray EA, Mcgillis WR (2003). Variation in surface turbulence and gas transfer velocity over a tidal cycle in a macro-tidal estuary. Estuaries.

[25] Borges AV (2004). Gas transfer velocities of CO_2_ in three European estuaries (Randers Fjord, Scheldt, and Thames). Limnol. Oceanogr..

[26] Munoz-Hincapie M, Morell JM, Corredor JE (2002). Increase of nitrous oxide flux to the atmosphere upon nitrogen addition to red mangroves sediments. Mar. Pollut. Bull..

[27] Srinivas K, Revichandran P, Maheswaran PA, Mohammed Ashraf TT, Nuncio M (2003). Propagation of tides in the Cochin estuarine system, southwest coast of India. Indian J. Geomar. Sci..

[28] Srinivas K, Revichandran C, Dinesh Kumar PK (2003). Statistical forecasting of met-ocean parameters in the Cochin estuarine system, southwest coast of India. Indian J. Geomar. Sci..

[29] Balachandran, K. K., Joseph, T., Nair, K. K. C., Nair, M. & Joseph, P. S. The complex estuarine formation of six rivers (Cochin backwaters system on westcoast of India)—Sources and distribution of trace metals and nutrients. In:APN/SASCOM/LOICZ Regional Workshop on Assessment of Material Fluxes To the Coastal Zone in South Asia and their Impacts. Sri Lanka National Committee of IGBP, Colombo, Sri Lanka, 359, http://drs.nio.org/drs/handle/2264/1340 (2002).

[30] Martin GD (2008). Freshwater influence on nutrient stoichiometry in a tropical estuary, southwest coast of India. Appl. Ecol. Environ. Res..

[31] Liu D, Zhong J, Zheng X, Fan C, Yu J, Zhong W (2018). N_2_O fluxes and rates of nitrification and denitrification at the sediment-water interface in Taihu Lake, China. Water.

[32] Luijn FV, Boers PCM, Lijklema L (1996). Comparison of denitrification rates in lake sediments obtained by the N_2_ flux method, the 15N isotope pairing technique and the mass balance approach. Water Res..

[33] Pfenning KS, McMahon PB (1997). Effect of nitrate, organic carbon, and temperature on potential denitrification rates in nitrate-rich riverbed sediments. J. Hydrol..

[34] Borges AV (2015). Globally significant greenhouse-gas emissions from African inland waters. Nat. Geosci..

[35] Marzadri A, Dee MM, Tonina D, Bellin A, Tank JL (2017). Role of surface and subsurface processes in scaling N_2_O emissions along riverine networks. Proc. Natl. Acad. Sci. U. S. A..

[36] Soued, C., del Giorgio, P. A. & Maranger, R. Nitrous oxide sinks and emissions in boreal aquatic networks in Quebec. *Nat. Geosci.* **9**(2), 116–120, https://www.x-mol.com/paperRedirect/68353 (2016).

[37] Hu MP, Chen DJ, Dahlgren RA (2016). Modeling nitrous oxide emission from rivers: A global assessment. Glob. Change Biol..

[38] Murray R, Erler DV, Rosentreter J, Wells NS, Eyre BD (2020). Seasonal and spatial controls on N_2_O concentrations and emissions in low-nitrogen estuaries: Evidence from three tropical systems. Mar. Chem..

[39] Ji QX, Babbin AR, Peng XF, Bowen JL, Ward BB (2015). Nitrogen substrate dependent nitrous oxide cycling in salt marsh sediments. J. Mar. Res..

[40] Punshon S, Moore RM (2004). Nitrous oxide production and consumption in a eutrophic coastal embayment. Mar. Chem..

[41] Corredor JE, Morell JM, Bauza J (1999). Atmospheric nitrous oxide fluxes from mangrove sediments. Mar. Pollut. Bull..

[42] Raymond PA (2012). Scaling the gas transfer velocity and hydraulic geometry in streams and small rivers. Limnol. Oceanogr. Fluids Environ..

[43] Alongi DM (2018). Impact of global change on nutrient dynamics in mangrove forests. Forests..

[44] Reef R, Feller IC, Lovelock CE (2010). Nutrition of mangroves. Tree Physiol..

[45] Muller D (2016). Nitrous oxide and methane in two tropical estuaries in a peat-dominated region of northwestern Borneo. Biogeosciences.

[46] Hasegawa T, Okino T (2004). Seasonal variation of denitrification rate in Lake Suwa sediment. Limnology.

[47] Myrstener, M., Jonsson, A. & Bergström, A. K. The effects of temperature and resource availability on denitrification and relative N_2_O production in boreal lake sediments. *J. Environ. Sci. (China).* 10.1016/j.jes.2016.03.003 27593275

[48] Strickland, J. D. H. & Parsons, T. R. *A Practical Handbook of Seawater Analysis*. 2nd edn. 310 (Fisheries Research Board of Canada, 1972).

[49] Grasshoff, K., Ehrhardt, M. & Kremling, K. *Methods of seawater analysis*. 2nd edn. 419 (Verlag Chemie, 1983).

[50] Garcia H, Gordon L (1992). Oxygen solubility in seawater: Better fitting equations. Limnol. Oceanogr..

[51] Grasshoff K, Ehrhardt M, Kremling K (1999). Methods of Seawater Analysis.

[52] David, A. R. Analysis of Total organic carbon. UMass Environmental Engineering Program (2012).

[53] Polunin NV (2001). Feeding relationships in Mediterranean bathyal assemblages elucidated by stable nitrogen and carbon isotope data. Mar. Ecol. Prog. Ser..

[54] McAuliffe C (1971). GC determination of solutes by multiple phase equilibrations. Chem. Tech..

[55] Liss, P. S. & Merlivat, L. Air-sea exchange rates: Introduction and synthesis, in the role of air-sea exchange in geochemical cycling. In (ed. Buat-Menard, P.) 113–127 (D Reidel, 1986) 10.1007/978-94-009-4738-2_5.

[56] Weiss RF, Price BA (1980). Nitrous oxide solubility in water and seawater. Mar. Chem..

[57] Rao GD, Rao VD, Sarma VVSS (2013). Distribution and air–sea exchange of Nitrous oxide in the Coastal Bay of Bengal during peak discharge period(southwest monsoon). Mar. Chem..

